# Codon optimized *Tol2* transposase results in increased transient expression of a *crystallin*-GFP transgene in zebrafish

**DOI:** 10.17912/micropub.biology.000268

**Published:** 2020-06-11

**Authors:** Allison S. Mackey, Priscilla S. Redd, April DeLaurier, C. Nathan Hancock

**Affiliations:** 1 University of South Carolina Aiken, Department of Biology and Geology, Aiken, SC; 2 University of Utah Bioscience Program, Salt Lake City, UT

**Figure 1:**
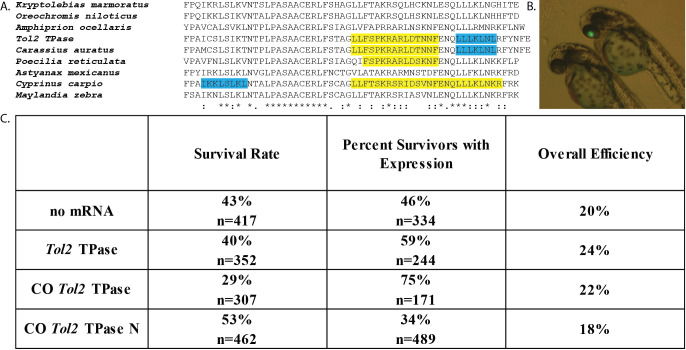
**Codon-optimization improves transgenesis in surviving fish** **A.** Alignment of the C-terminal region of the *Tol2* TPase protein and its homologs. Predicted NLS sequences are highlighted in yellow, predicted NES sequences are highlighted in blue. **B.** Image of zebrafish embryos 2 days post fertilization (dpf) after injection with pDestTol2pACryGFP and CO *Tol2* TPase mRNA. Right embryo is positive for eye-localized eGFP expression. **C.** Results from embryo co-injection of pDestTol2pACryGFP with four different mRNA treatments. Survival rate is the proportion of fish that survived 2 dpf for a subset of the total injections (n = number of injections for which survival was measured). Overall efficiency was calculated by multiplying the survival rate by the percent of surviving fish that showed eye localized eGFP expression.

## Description

Type II transposable elements (TEs) are segments of DNA that can be mobilized within the genome through the action of transposase (TPase) proteins (Craig 2002). In general, the rate at which TPase proteins bind the terminal sequences of the elements to form a functional transposition complex determines the relative mobility of the element (Mizuuchi *et al.* 1992; Zayed *et al.* 2004; Zhang *et al.* 2001). The rate of transposition complex formation is thus determined by the concentration of functional TPase protein, the number of TE sequences present, and the localization of the TPase proteins within the cell. Several studies suggest that transposition complex formation is regulated by access to the nucleus, as alteration of nuclear localization signals (NLS) and nuclear export signals (NES) influences transposition (Hancock *et al.* 2010; Payero *et al.* 2016; Ramakrishnan *et al.* 2019). Zebrafish studies often involve the integration of DNA sequences (i.e. overexpression cassettes) into embryos, leading to transient or germ line expression (Nusslein-Volhard and Dahm 2002). Inserting transgenes between TE sequences and providing a TPase protein source has been shown to increase transgenesis efficiency across multiple model organisms (Ding *et al.* 2005; Ivics and Izsvák 2004; Munoz-Lopez and Garcia-Perez 2010; Zayed *et al.* 2004). The *Tol2* TE from Medaka fish (*Oryzias latipes*) has been developed as a means to improve zebrafish transgenesis, including both transient and germline integration (Kawakami 2007; Koga *et al.* 1996; Kwan *et al.* 2007; Ni *et al.* 2016). *Tol2*-mediated transgenesis isinduced by co-injecting *Tol2* TPase mRNA together with a *Tol2* terminal sequence-flanked construct into 1-4 cell stage zebrafish embryos (Kawakami 2007; Kwan *et al.* 2007; Ni *et al.* 2016). Previous reports indicated that addition of the *Tol2* TPase mRNA (expressed from pCS2FA) produced about a 3-fold increase in *Tol2* flanked transgene expression compared to control (Kwan *et al.* 2007). Another study demonstrated that a zebrafish codon optimized version of *Tol2* TPase led to successful germline transmission, but no direct comparison of efficiency was reported (Suster *et al.* 2011). The goal of this study was to explore the extent that efficiency of *Tol2*-mediated transgene expression could be improved by codon-optimizing the *Tol2* TPase gene for zebrafish or altering a detected NES and NLS in the *Tol2* TPase (Figure 1A). We hypothesized that increasing the translation efficiency (Gustafsson *et al.* 2004) and access to the nucleus would potentially improve transposition complex formation in transience. Analysis of the *Tol2* TPase codon usage showed that it contained 19 codons (TTA, CTA, and TCG) that are rarely used by zebrafish (Nakamura *et al.* 2000). Codon optimization resulted in the development of a CO *Tol2* TPase construct that more appropriately matched the zebrafish-specific codon bias, without altering the amino acid sequence (Zhou *et al.* 2016). In the CO *Tol2* TPase N construct, the NES in the *Tol2* TPase (Figure 1A) was removed by changing L641A and L643A and the existing NLS (PKRARLD, NLS score=8.5) was strengthened by changing it to PKKKRKV (NLS score=13) (Dingwall and Laskey 1991; Kosugi *et al.* 2009).

Injection of 1-4 cell-stage zebrafish embryos with pDestTol2pACryGFP showed a significantly increased frequency of eGFP expression in the eye (i.e. Figure 1B) when co-injected with wild type *Tol2* TPase mRNA compared to no mRNA (Figure 1C, p=0.0024). This is below the reported 3-fold increased frequency of expression previously reported using non-codon optimized *Tol2* (Kwan *et al.* 2007). We observed a significantly increased frequency of eye eGFP expression when we used the codon optimized (CO *Tol2* TPase) mRNA (Figure 1C, vs. no mRNA p=<0.0001, vs. WT TPase p=0.0008). These results suggest that the codon optimized version of the mRNA is translated more effectively in embryos, leading to an overall higher concentration of *Tol2* TPase protein. In contrast, the NES and NLS altered version (CO *Tol2* TPase N) showed decreased expression frequency compared to all other groups (vs. no mRNA p=0.0013; vs. WT *Tol2* TPase p<0.0001; vs. CO *Tol2* TPase p<0.0001). This suggests that the changes made to the NES and NLS may have disrupted overall protein function of the CO *Tol2* TPase N protein (Figure 1C).

When both the expression frequencies and survival rates were used to calculate overall transgene expression efficiency, we observed that all treatments resulted in about 20% of the injected eggs producing eGFP-expressing embryos surviving to 2 dpf (Figure 1C). An inverse relationship was observed between the survival frequency and the frequency of eGFP expression at 2 dpf. We observed that significantly less fish developed properly with the higher activity CO *Tol2* TPase mRNA compared to no mRNA (p = 0.0002) and the WT *Tol2* TPase mRNA (p=0.0033) (Figure 1C). This suggests that genome disruption potentially caused by transgene insertion may be an important limit on transgenesis rates.

The high frequency of eGFP expression observed in this study for the no mRNA control suggests that a high percentage of fluorescence observed was not TPase mediated. In addition, our results suggest that the addition of *Tol2* mRNA does not drastically increase the overall rate of transient expression of transgenes (total number of surviving transgenic fish/ embryos injected). However, there are significant benefits to the *Tol2* system, as a greater fraction of fish that survive show transient transgene expression, potentially leading to easier screening for expressing embryos. Our results suggest that co-injecting constructs with the CO *Tol2* TPase mRNA (available through Addgene #133032) is a significant improvement over the existing technology. This codon optimized *Tol2* has the potential to increase the yield of transient expression and should be tested for its ability to induce heritable transgenesis. Although this study did not examine heritable transgenesis, the next step in this investigation would be to examine the rates of integration of the pDestTol2pACryGFP transgene in fish injected with each mRNA.

## Methods

Sequence analysis

The *Tol2* TPase sequence (GenBank: BAA87039.1) was used in NCBI’s Basic Local Alignment Search Tool (BLAST) to obtain homologs. The alignment was performed using EMBL-EBI Clustal Omega Multiple Sequence Alignment tool (Sievers *et al.* 2011). NLS and NES sequences were identified using cNLS Mapper (Kosugi *et al.* 2009) and NetNES 1.1 Server (La Cour *et al.* 2004), respectively.

Construction of novel plasmids

IDT’s codon optimization tool (https://www.idtdna.com/codonopt) was used to generate a novel sequence that altered most of the codons, removing TTA, CTA, and TCG codons. The two new versions of *Tol2* TPase were synthesized as IDT gBlocks®, cloned into the *Bam*HI and *Xba*I sites of pCS2FA plasmid (Kwan *et al.* 2007), and sequence verified. Plasmid DNA was purified using Zyppy™ Plasmid Miniprep and quantitated by Qubit® dsDNA BR Assay.

mRNA preparation

Plasmids were linearized with *Not*I-HF, column purified using the Zymo DNA Clean and Concentrator™ -5 kit and quantitated by Qubit® dsDNA BR Assay. *In vitro* transcription was performed on 1 µg of DNA using the Ambion mMessage mMachine™ SP6 Kit. After the transcription reaction, the product was treated with TURBO DNase and cleaned using the Nucleospin® RNA II columns. Gel electrophoresis was used to confirm for the presence of mRNA and the concentration was determined by Qubit® RNA BR Assay.

Injection of mRNA and screening for GFP expression in zebrafish

A previously established transgenesis assay (Berger and Currie 2013) in which pDestTol2pACryGFP produces eye specific *eGFP* expression from the *crystallin* (cryaa) promoter (Kurita *et al.* 2003) was used. pDestTol2pACryGFP was a gift from Joachim Berger & Peter Currie (Addgene plasmid # 64022 ; http://n2t.net/addgene:64022 ; RRID:Addgene_64022). eGFP fluorescence was detected by fluorescence microscopy 2 dpf (Figure 1B). Adult wild-type AB (ZFIN) zebrafish were mated and injections were performed on resulting embryos within one hour post fertilization (1-4 cell stage). Each embryo was injected with 25 pg of mRNA and 30 pg of pDestTol2pACryGFP DNA as described (Kwan *et al.* 2007). Two treatments were injected each day until 6 replicate injections of each mRNA were achieved. The order of treatments was continually altered to prevent injection timing bias. Screening of the embryos took place at 2 dpf on either an Olympus CKX41 Inverted Microscope or an Olympus SZX12 Fluorescence Stereo Microscope.

**Statistical Analysis**

eGFP expression and survival data were pooled and analyzed using a contingency table. A χ^2^ analysis was used to identify significance and multiple comparisons were conducted using Fisher’s exact test (SAS version 9.4). The null hypothesis was that the proportion observed was independent of the treatment.
